# Tissue-Specific Autoantibodies Improve Diagnosis of Primary Sjögren's Syndrome in the Early Stage and Indicate Localized Salivary Injury

**DOI:** 10.1155/2019/3642937

**Published:** 2019-05-07

**Authors:** Yuebo Jin, Jing Li, Jiali Chen, Miao Shao, Ruijun Zhang, Yichen Liang, Xia Zhang, Xiaoying Zhang, Qin Zhang, Fangting Li, Yaobin Cheng, Xiaolin Sun, Jing He, Zhanguo Li

**Affiliations:** ^1^Department of Rheumatology & Immunology, Peking University People's Hospital, Beijing 100044, China; ^2^Beijing Key Laboratory for Rheumatism Mechanism and Immune Diagnosis (BZ0135), China; ^3^Department of Rheumatology and Immunology, First Affiliated Hospital of Xiamen University, Xiamen 361003, China; ^4^Department of Ophthalmology, Peking University People's Hospital, Beijing 100044, China; ^5^College of Optometry, Peking University Health Science Center, China; ^6^Peking-Tsinghua Center for Life Sciences, Beijing, China

## Abstract

Primary Sjögren's syndrome (pSS) is a chronic autoimmune disease characterized by lymphocytic infiltration of exocrine glands. Due to the absence of specific clinical manifestations and biomarkers in the early stage, pSS is generally underrecognized. To elucidate the role of the tissue-specific autoantibodies (TSAs), i.e., anti-CA6, anti-SP1, and anti-PSP antibodies, we enrolled 137 pSS patients, 32 secondary Sjögren's syndrome (sSS) patients, and 127 healthy controls (HCs), whose serum and saliva samples were collected. TSA levels were detected by ELISA, and the clinical and laboratory data was reviewed from the medical records. The analysis results showed the following: (1) Compared to HCs, the serum IgA levels of anti-CA6, anti-SP1 and anti-PSP were significantly higher in pSS as well as in sSS patients, and anti-CA6 IgG was also notably higher in pSS patients. (2) The positivity of anti-CA6, anti-PSP and all the three antibodies together were significantly increased in anti-SSA-negative pSS patients. (3) The average IgM levels of anti-CA6 and anti-SP1 decreased as the disease duration extended. (4) The anti-CA6-positive patients have significantly higher levels of serum IgA, while the anti-PSP-positive group has a notably higher serum IgM level. (5) Another autoantibody specific to the salivary glands, anti-*α*-fodrin antibody, was elevated in TSA-positive patients, especially in the anti-CA6-positive group. (6) Preliminary detection of saliva TSAs showed that all the IgG levels of these three antibodies increased significantly in pSS patients. In conclusion, TSAs improve diagnosis of pSS in the early stage, especially in anti-SSA-negative patients, and their tissue-specific nature indicates localized salivary injury, which deserves further studies to clarify the mechanism.

## 1. Introduction

Primary Sjögren's syndrome (pSS) is an autoimmune disease featured by chronic lymphocytic infiltration of exocrine glands (notably lacrimal and salivary glands) as well as systemic impairments, which often progresses insidiously but could also be life-threatening if not diagnosed and treated in a timely manner [1]. As one of the most common autoimmune diseases in adults [2], the prevalence rate of pSS in China was estimated as 0.33-0.77% [3] and a population of nearly 10 million is affected. Unfortunately, because of the lack of specific clinical manifestations and biomarkers in the early stage, pSS patients were usually underrecognized [4], which underlines the importance of early identification of pSS patients before irreversible damage to the organs and tissues occurs.

The onset of pSS is highly associated with abnormal B cell activation and the production of excessive autoantibodies. At present, the most commonly applied biomarkers in clinical practice, such as anti-SSA antibody, anti-SSB antibody, rheumatoid factor (RF), and ANA, bear their own limitations, especially in the early stage of disease [5]. Previous studies have reported that anti-CA6, anti-SP1, and anti-PSP antibodies, i.e., the tissue-specific autoantibodies (TSAs), have shown significance in the early diagnosis of pSS in both animal models and patients [6]. However, there still lack sufficient clinical studies. Based upon the tissue-specific nature of these antibodies, we hypothesized that TSAs could improve diagnosis of pSS in the early stage, especially in those anti-SSA-negative patients, and could indicate localized salivary injury. Our research focused on the early diagnosis value of these autoantibodies and attempted to preliminarily explore the underlying mechanisms through clinical and laboratory data.

## 2. Materials and Methods

### 2.1. Patients and Controls

We retrospectively analyzed the data of 137 patients with primary SS and 32 SLE patients with secondary SS (sSS) who had been admitted to Peking University People's Hospital between January 2016 and December 2017. All SS patients were diagnosed based on the revised US-EURO classification criteria for SS (2002) or ACR classification criteria (2012). SLE patients were diagnosed according to the 1997 revised classification criteria of ACR or the 2017 SLICC classification criteria. And patients with other diseases such as RA and diabetes were excluded. One hundred and twenty seven healthy controls (HCs) were selected from age- and sex-matched disease-free subjects who underwent routine medical examinations at our hospital.

This study was approved by the Medical Ethics Committee of Peking University People's Hospital. All the subjects provided their written informed consents before the samples were obtained. Patient consent was obtained according to the Declaration of Helsinki.

### 2.2. Blood and Saliva Samples

The serum samples of 137 pSS patients, 32 sSS patients, and 127 HCs were collected and preserved at -80°C before tests. Their clinical and laboratory data were reviewed. Among them, saliva samples of 12 pSS patients and 24 HCs were also obtained using a specialized device—RNAPro•SAL™ Oral Specimen Collection System (produced by Oasis Diagnostics® Corporation)—following the instruction and centrifuged. Further antibody detection was done in 24 hours.

### 2.3. Enzyme-Linked Immunosorbent Assay (ELISA)

Autoantibodies to CA6, SP1, and PSP were determined using ELISA kits (Immco Diagnostics Inc., Buffalo, NY). Serum samples were diluted 100 times according to the instruction of the ELISA kit, and the saliva samples were diluted 10 times according to our pilot experiments. Following the instruction, the results were expressed in ELISA units per milliliter (EU/ml) and defined as positive for a value ≥ 20 EU/ml or negative for a value < 20 EU/ml in the serum.

### 2.4. Clinical and Laboratory Evaluation

Clinical and laboratory data were reviewed retrospectively from the medical records of pSS patients, including demographic characteristics (among them, 104 patients are able to provide the exact disease durations) and extensive serological analysis. RF was analyzed by the nephelometric method (Beckman Coulter, USA), and anti-*α*-fodrin antibody was tested by ELISA (Shanghai Kexin Biotech Co., China). ANA was detected by indirect immunofluorescence, while anti-SSA and anti-SSB antibodies were detected using a double immunodiffusion kit (EUROIMMUN, Germany) according to the manufacturer's instructions. The results of routine blood tests and serological features such as C-reactive protein (CRP), erythrocyte sedimentation rate (ESR), immunoglobulins, C3 and C4 levels were also collected.

### 2.5. Statistical Analysis

Comparisons were tested for statistical significance using the Student *t*-test, the Mann-Whitney *U* test, or the chi-squared test, as appropriate. Multiple logistic regressions were performed with antibody production as the dependent variable. All statistical analysis were performed with SPSS 16.0. *P* values < 0.05 were considered statistically significant.

## 3. Results

### 3.1. General Information of the pSS Patients Enrolled

As shown in Table 1, our study enrolled 137 pSS patients and 129 (96.3%) of them were female. The average age was 57.6 ± 12.6 years, and the average disease duration was 10.9 ± 9.5 years.

### 3.2. Levels of Serum TSAs in SS Patients Compared to HCs

**Figure 1:**
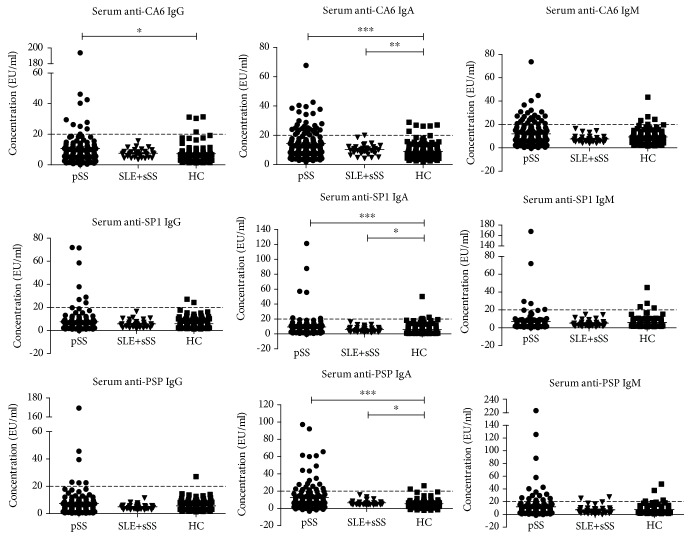
Levels of serum TSAs in SS patients compared to those in HCs. The serum IgA levels of anti-CA6, anti-SP1, and anti-PSP were significantly higher in both pSS and sSS patients. Serum anti-CA6 IgG was remarkably higher in pSS patients. Others presented no significant difference. Dotted lines denote the cutoff for the positive value, 20 EU/ml. (^∗^*P* < 0.05, ^∗∗^*P* < 0.01, ^∗∗∗^*P* < 0.001).

Figure 1 illustrates the IgG, IgA, and IgM levels of anti-CA6, anti-SP1, and anti-PSP antibodies in SS patients and HCs. Compared to HCs, the serum IgA levels of anti-CA6, anti-SP1, and anti-PSP were significantly higher in both pSS and sSS patients (*P* < 0.05). Serum anti-CA6 IgG was remarkably higher in pSS patients (*P* < 0.05). Others presented no significant difference.

### 3.3. The Positive Rates of Serum TSAs in pSS Patients in Comparison with HCs

According to the instruction of the ELISA kit, a value ≥ 20 EU/ml was determined as positive of the antibodies. We defined the positivity of the antibodies as at least one of the IgG, IgM, or IgA ≥ 20 EU/ml. As shown in Table 2, the positivities of anti-CA6, anti-PSP, and TSA (anti-CA6, anti-SP1, and anti-PSP three together) were significantly higher in pSS patients than in HCs (*P* < 0.05).

Then, we focused on the positivity of TSAs in anti-SSA-, anti-SSB-, and ANA-negative patients.  Table 3 reveals the significance of anti-PSP antibody and TSA especially in anti-SSA-negative patients (*P* < 0.05). It could help to recognize pSS patients without the classic anti-SSA antibody.

### 3.4. The Relationship of Serum Levels of TSAs and Disease Duration in pSS Patients

**Figure 2:**
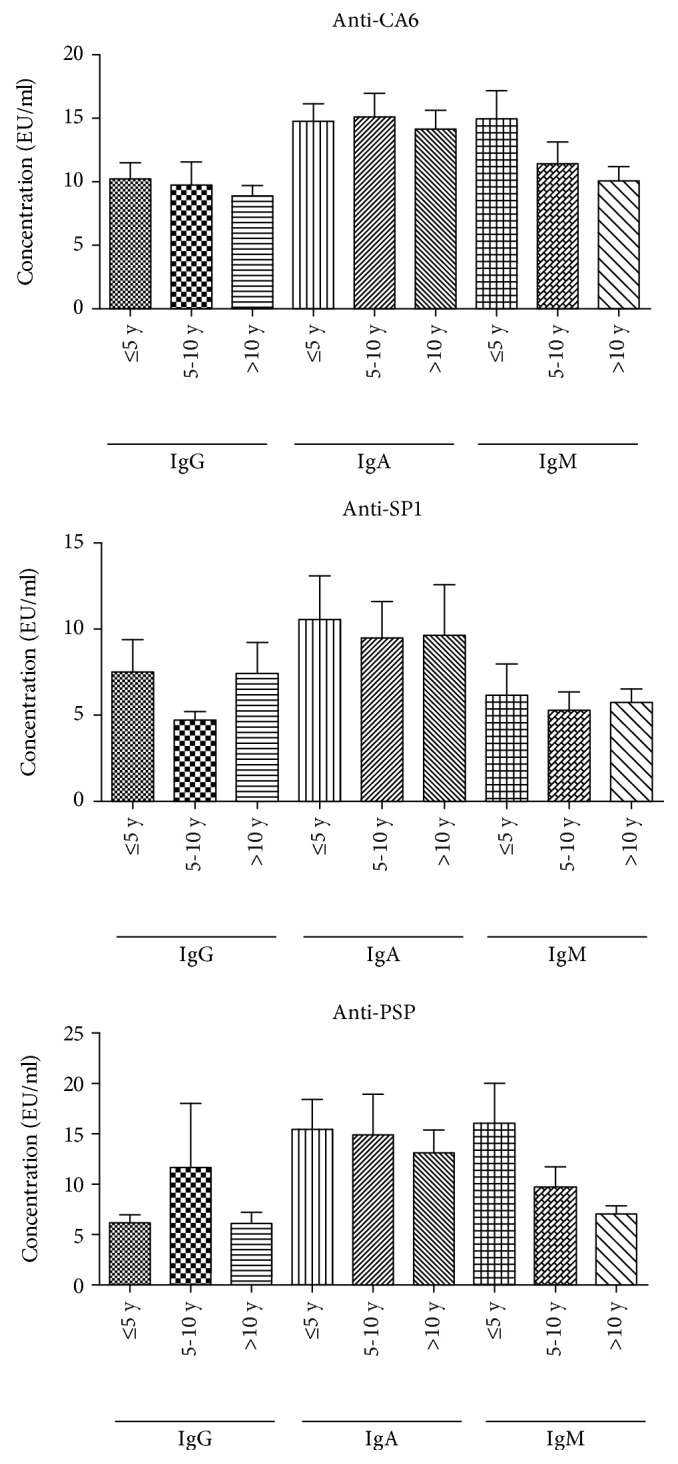
The concentrations of TSAs in different groups of disease duration. The average IgM levels of anti-CA6 and anti-PSP antibodies decreased as the disease duration extended.

We divided the pSS patients into 3 groups according to their disease durations: ≤5 years, 5-10 years, and >10 years. The concentrations of serum TSAs in different groups are shown in Figure 2. The average IgM levels of anti-CA6 and anti-PSP antibodies decreased as the disease duration extended.

### 3.5. The Comparison of Clinical and Laboratory Data in TSA-Positive and TSA-Negative pSS Patients

The clinical and laboratory data were analyzed, and we compared the pSS patients with positive and negative antibodies (Table 4). In serum anti-CA6-positive patients, the level of serum IgA was significantly higher than that in anti-CA6-negative patients. The anti-PSP-positive group revealed a notably higher serum IgM level. Interestingly, the level of anti-*α*-fodrin antibody, which is also an autoantibody specific to the salivary gland, was elevated in all the TSA-positive patients, especially in the anti-CA6-positive group.

### 3.6. A Preliminary Exploration of Saliva TSA Levels in pSS Patients

**Figure 3:**
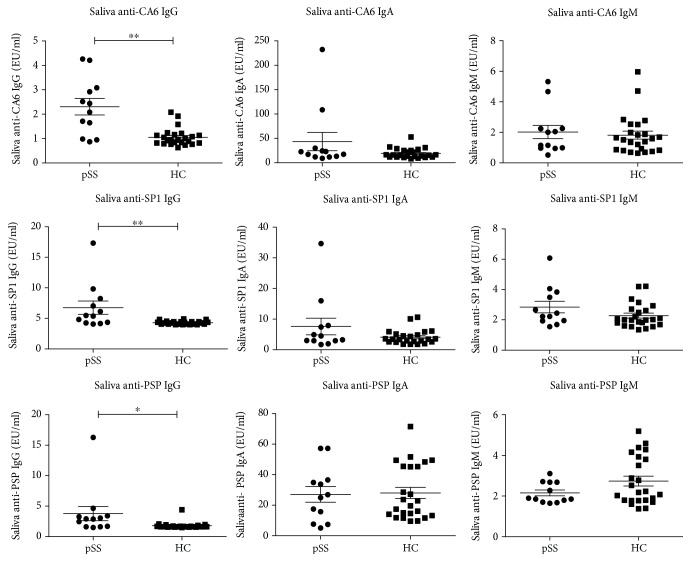
Comparison of saliva TSA levels in pSS patients and HCs. All the IgG levels of the three antibodies were significantly higher in the saliva of pSS patients (^∗^*P* < 0.05, ^∗∗^*P* < 0.01).

To explore the role of TSAs in salivary glands and in the mechanism of pSS, we detected a few patients' salivary TSA levels and compared them with the TSA levels of HCs. Interestingly, all the IgG levels of the three antibodies increased significantly in the saliva of pSS patients (Figure 3). This may indicate the role of TSAs in salivary-localized pathology of pSS.

Further correlation analysis of serum and saliva TSA levels showed that there was no direct relationship between the antibody levels in the serum and in the saliva in pSS patients (Table 5).

## 4. Discussion

Primary Sjögren's syndrome (pSS) is a chronic autoimmune disease without distinct and specific manifestations and biomarkers, especially in the early stage. Since pSS is characterized by B cell activation, autoantibodies play a crucial role in the development of the disease [7]. So far, the most widely accepted autoantibodies associated with pSS are anti-SSA and anti-SSB antibodies, ANA, and RF [8, 9]. Among them, the anti-SSA antibody is reserved as the only meaningful serum biomarker in the most recent criteria of pSS in 2016 [10]. However, as the SSA antigen is distributed in all the somatic cells, the presence of the anti-SSA antibody may infer systemic injury and a relatively late stage of the disease. Therefore, in some cases, the diagnosis established on anti-SSA and other commonly used autoantibodies such as anti-SSB and ANA may lead to the underrecognition of pSS in the so-called “seronegative” patients or in the very early stage, which is exactly the key diagnosis phase when damage to organs has not yet appeared.

The anti-CA6, anti-SP1, and anti-PSP antibodies were first identified from the IL-14*α*-transgenic pSS animal model [6, 11], and all of their antigens were located in the salivary glands, hence the name “tissue-specific autoantibodies”. Interestingly, in this animal model, TSAs appeared even earlier than anti-SSA and anti-SSB antibodies. Soon, these organ-specific autoantibodies drew attention and inspired discussion on the criteria based on the traditional autoantibodies [12, 13]. Further investigations exhibited the significance of TSAs in both pSS animal models and patients, especially in the early stage of the disease [6, 14]. Just as recent researches reported, autoantibodies may present years before symptom onset in pSS [15, 16], confirming the precursory role of autoantibodies, very likely including TSAs. Furthermore, several clinical studies have revealed that TSAs are meaningful in distinguishing pSS patients from dry eye populations [17–19] and appear to be a useful tool to recognize pSS patients who are so-called “seronegative” or in the early stage [20].

Our data showed that TSAs could distinguish pSS patients from healthy controls in accord with previous studies [17, 21]. Moreover, they were also of good significance in anti-SSA-negative patients in accord with other studies [14]. And as the disease developed, the levels of TSAs tended to decrease. Besides, the TSA levels were positively correlated with another salivary tissue-specific antibody, anti-*α*-fodrin antibody [22], and the IgA levels of TSAs in patients' saliva were independently elevated. These may all be related to the antigens' localized nature. Unlike the well-acknowledged autoantibodies such as anti-SSA, and other novel autoantibodies such as antivimentin antibody [23] and anti-calponin-3 antibodies [24], whose target antigens spread in various tissues, the antigens of TSAs are localized in the salivary glands. Therefore, we hypothesized that, in the early stage of pSS, salivary gland tissues full of localized exposing autoantigens were still abundant, thus triggering and stimulating the abnormal activation of the autoimmune T cell and B cell to produce more tissue-specific antibodies, which in turn damage the salivary gland tissues and other organs. As the disease duration extended, the salivary glands were injured and atrophied, resulting in the decrease of TSAs, while antibodies to SSA emerged and became prominent in the later stage. Just like the other antibodies [25], it is possible that TSAs may be related to the clinical phenotypes of pSS, which demands further study.

There still lack exact mechanism studies to elucidate the role of these TSAs in pSS. For example, the specific antigen epitopes of these antigens still remain unknown. More in vivo and in vitro experiments are ongoing to reveal the pathogenesis of the TSAs from different aspects.

In conclusion, this study showed that TSAs may be an effective tool in recognizing pSS patients in the early stage, especially in anti-SSA-negative patients. The underlying mechanism may be related to their localized nature. More researches need to be carried out to further clarify the pathological mechanism of TSAs in pSS. There is hope that TSAs could help to improve the diagnosis and prognosis in future clinical practice.

## Figures and Tables

**Table 1:** General information of the pSS patients enrolled.

General information	Result
Sex (M/F)	8/129
Age (years, mean ± SD)	57.6 ± 12.6
Disease duration (years, mean ± SD)	10.9 ± 9.5

**Table 2:** Comparison of the positivity of TSAs of the enrolled pSS patients and HCs.

	pSS (*n* = 137)	HC (*n* = 127)	*P*
Anti-CA6	51 (37.2%)	14 (11.0%)	<0.001
Anti-SP1	14 (10.2%)	9 (7.1%)	0.367
Anti-PSP	35 (25.5%)	7 (5.5%)	<0.001
TSA	62 (45.3%)	26 (20.5%)	<0.001

**Table 3:** Comparison of the positivity of TSAs in patients with and without anti-SSA, anti-SSB, and ANA.

Antibodies	Anti-SSA+ (*n* = 108)	Anti-SSA- (*n* = 29)	*P*	Anti-SSB+ (*n* = 57)	Anti-SSB- (*n* = 80)	*P*	ANA+ (*n* = 118)	ANA- (*n* = 19)	*P*
Anti-CA6+	37 (34.3%)	14 (48.3%)	0.166	20 (35.1%)	31 (38.8%)	0.662	44 (37.3%)	7 (36.8%)	0.970
Anti-SP1+	10 (9.3%)	4 (13.8%)	0.495	7 (12.3%)	7 (8.8%)	0.501	12 (10.2%)	2 (10.5%)	1.000
Anti-PSP+	21 (19.4%)	14 (48.3%)	0.002^∗^	13 (22.8%)	22 (27.5%)	0.960	29 (24.6%)	6 (31.6%)	0.573
TSA+	44 (40.7%)	18 (62.1%)	0.04^∗^	23 (40.4%)	39 (48.8%)	0.986	53 (44.9%)	9 (47.4%)	0.728

^∗^ *P* < 0.05.

**Table 4:** The comparison of clinical and laboratory data in TSA-positive and TSA-negative pSS patients.

	Anti-CA6+ (*n* = 51)	Anti-CA6- (*n* = 86)	*P*	Anti-SP1+ (*n* = 14)	Anti-SP1- (*n* = 123)	*P*	Anti-PSP+ (*n* = 35)	Anti-PSP- (*n* = 102)	*P*	TSA+ (*n* = 62)	TSA- (*n* = 75)	*P*
Female	49 (96.1%)	80 (93.0%)	0.710	13 (92.9%)	116 (94.3%)	0.588	33 (94.3%)	96 (94.1%)	1.000	59 (95.2%)	70 (93.3%)	0.729
Age (years)	58.8 ± 2.0	56.9 ± 1.3	0.399	60.2 ± 2.7	57.3 ± 1.2	0.422	59.0 ± 2.2	57.2 ± 1.2	0.452	56.7 ± 2.9	57.1 ± 1.3	0.906
Duration (years)	12.0 ± 1.9	10.8 ± 1.1	0.553	14.7 ± 5.3	10.8 ± 0.9	0.504	13.3 ± 2.5	10.5 ± 0.9	0.307	10.0 ± 1.7	10.6 ± 1.1	0.754
WBC (10^9^/L)	5.5 ± 0.3	5.4 ± 0.3	0.904	5.3 ± 0.5	5.5 ± 0.2	0.797	5.4 ± 0.4	5.5 ± 0.2	0.915	5.7 ± 0.4	5.4 ± 0.3	0.476
Hb (g/L)	120.1 ± 1.9	118.7 ± 2.4	0.699	116.6 ± 5.2	119.5 ± 1.8	0.597	118.0 ± 3.7	119.7 ± 1.8	0.233	117.9 ± 3.3	119.7 ± 2.4	0.675
PLT (10^9^/L)	159.6 ± 10.9	163.7 ± 8.4	0.765	163.5 ± 19.9	162.0 ± 7.1	0.946	158.8 ± 15.0	163.3 ± 7.3	0.347	165.5 ± 17.2	164.1 ± 8.3	0.937
CRP (mg/L)	7.7 ± 2.8	8.3 ± 1.9	0.863	7.0 ± 4.6	8.2 ± 1.7	0.817	10.5 ± 4.1	7.1 ± 1.5	0.346	8.4 ± 3.4	7.9 ± 1.9	0.892
ESR (mm/h)	28.7 ± 4.2	27.6 ± 3.1	0.340	44.4 ± 10.4	28.7 ± 2.5	0.167	37.6 ± 6.0	27.6 ± 2.6	0.131	28.1 ± 4.6	27.4 ± 3.2	0.904
*γ*G (%)	23.9 ± 1.3	22.7 ± 0.9	0.472	24.8 ± 1.6	23.0 ± 0.8	0.472	25.2 ± 1.6	22.4 ± 0.8	0.089	22.1 ± 1.7	22.6 ± 0.9	0.801
IgG (g/L)	19.2 ± 1.4	18.4 ± 1.0	0.633	20.5 ± 1.9	18.5 ± 0.9	0.449	20.0 ± 1.7	18.3 ± 0.9	0.355	17.7 ± 1.8	18.4 ± 1.1	0.726
IgA (g/L)	4.6 ± 0.5	3.3 ± 0.3	0.013^∗^	4.9 ± 0.6	3.6 ± 0.3	0.149	4.1 ± 0.4	3.6 ± 0.3	0.416	4.1 ± 0.8	3.3 ± 0.3	0.280
IgM (g/L)	1.8 ± 0.2	1.4 ± 0.1	0.055	2.4 ± 0.5	1.2 ± 0.1	0.099	2.2 ± 0.3	1.3 ± 0.1	0.005^∗^	1.4 ± 0.2	1.2 ± 0.1	0.480
C3 (g/L)	0.94 ± 0.04	0.92 ± 0.02	0.759	0.97 ± 0.08	0.92 ± 0.02	0.473	0.93 ± 0.05	0.93 ± 0.02	0.918	0.91 ± 0.04	0.93 ± 0.03	0.794
C4 (g/L)	0.19 ± 0.01	0.19 ± 0.00	0.729	0.21 ± 0.03	0.19 ± 0.01	0.470	0.19 ± 0.08	0.19 ± 0.07	0.997	0.20 ± 0.01	0.19 ± 0.01	0.804
RF (IU/mL)	135.2 ± 18.9	110.8 ± 13.5	0.285	152.9 ± 37.4	116.1 ± 11.5	0.313	144.3 ± 22.7	111.5 ± 12.5	0.195	133.8 ± 24.4	103.4 ± 14.2	0.266
Anti-*α*-fodrin (U/ml)	19.8 ± 3.9	10.6 ± 1.4	0.031^∗^	40.9 ± 15.7	9.3 ± 1.0	0.099	24.0 ± 6.4	11.0 ± 0.9	0.054	17.4 ± 2.2	8.5 ± 0.8	0.001^∗^
ESSDAI	4.3 ± 0.3	4.8 ± 0.4	0.373	4.3 ± 0.8	4.6 ± 0.3	0.738	4.7 ± 0.5	4.6 ± 0.3	0.915	4.5 ± 0.4	4.7 ± 0.4	0.646

^∗^ *P* < 0.05.

**Table 5:** Correlation analysis of serum and saliva TSA levels.

Antibodies	*R*	*P*
Anti-CA6		
IgG	-0.287	0.365
IgM	0.146	0.651
IgA	0.450	0.224
Anti-SP1		
IgG	0.500	0.170
IgM	-0.252	0.429
IgA	0.117	0.764
Anti-PSP		
IgG	-0.025	0.949
IgM	0.295	0.352
IgA	0.392	0.208

## Data Availability

The data used to support the findings of the current study are available from the corresponding authors upon reasonable request.
